# Letter to the editor on: Hornerin deposits in neuronal intranuclear inclusion disease: direct identification of proteins with compositionally biased regions in inclusions by Park et al*.* (2022)

**DOI:** 10.1186/s40478-023-01706-7

**Published:** 2024-01-02

**Authors:** Huihui Luo, Emil K. Gustavsson, Hannah Macpherson, Natalia Dominik, Kristina Zhelcheska, Kylie Montgomery, Claire Anderson, Wai Yan Yau, Stephanie Efthymiou, Chris Turner, Michael DeTure, Dennis W. Dickson, Keith A. Josephs, Tamas Revesz, Tammaryn Lashley, Glenda Halliday, Dominic B. Rowe, Emily McCann, Ian Blair, Andrew J. Lees, Pentti J. Tienari, Anu Suomalainen, Laura Molina-Porcel, Gabor G. Kovacs, Ellen Gelpi, John Hardy, Matti J. Haltia, Arianna Tucci, Zane Jaunmuktane, Mina Ryten, Henry Houlden, Zhongbo Chen

**Affiliations:** 1 Department of Neuromuscular Disease, Queen Square Institute of Neurology, University College London (UCL), London, UK; 2 Department of Genetics and Genomic Medicine, Great Ormond Street Institute of Child Health, University College London, London, UK; 3 NIHR Great Ormond Street Hospital Biomedical Research Centre, University College London, London, UK; 4 Department of Neurodegenerative Disease, Queen Square Institute of Neurology, UCL, London, UK; 5 The Perron Institute for Neurological and Translational Science, Perth, Australia; 6 The National Hospital for Neurology and Neurosurgery, Queen Square, London, UK; 7 Department of Neuroscience, Mayo Clinic, Jacksonville, FL USA; 8 Neurodegenerative Research Group, Mayo Clinic, Rochester, MN USA; 9 Queen Square Brain Bank, Department of Clinical and Movement Neurosciences, Queen Square Institute of Neurology, UCL, London, UK; 10 Neuroscience Research Australia, Sydney, Australia; 11 School of Medical Sciences, Faculty of Medicine, University of New South Wales, Sydney, Australia; 12 Brain and Mind Centre, Sydney Medical School, The University of Sydney, Sydney, Australia; 13 Centre for Motor Neuron Disease Research, Department of Biomedical Sciences, Faculty of Medicine and Health Sciences, Macquarie University, Sydney, NSW Australia; 14 Reta Lila Weston Institute, UCL Queen Square Institute of Neurology, Wakefield Street, London, UK; 15 Department of Neurology, Helsinki University Hospital, Helsinki, Finland; 16 Translational Immunology Research Program, Faculty of Medicine, University of Helsinki, Helsinki, Finland; 17 Research Programs Unit, Stem Cells and Metabolism, University of Helsinki, 00290 Helsinki, Finland; 18 Neuroscience CenterHiLife, University of Helsinki, 00290 Helsinki, Finland; 19 HUSlab, Helsinki University Hospital, 00290 Helsinki, Finland; 20 Alzheimer’s Disease and Other Cognitive Disorders Unit. Neurology Service, Hospital ClínicFundació de Recerca Clínic Barcelona-Institut d’Investigacions Biomediques August Pi I Sunyer (FRCB-IDIBAPS), University of Barcelona, Barcelona, Spain; 21 Neurological Tissue Bank of the Hospital Clinic-IFRCB-IDIBAPS-Biobank, Barcelona, Spain; 22 Tanz Centre for Research in Neurodegenerative Disease, University of Toronto, Toronto, Canada; 23 Division of Neuropathology and Neurochemistry, Department of Neurology, Medical University of Vienna, Vienna, Austria; 24 Dementia Research Institute at UCL, Queen Square Institute of Neurology, UCL, London, UK; 25 NIHR University College London Hospitals Biomedical Research Centre, London, UK; 26 Institute for Advanced Study, The Hong Kong University of Science and Technology, Hong Kong SAR, China; 27 Department of Pathology, Faculty of Medicine, University of Helsinki, Helsinki, Finland; 28 William Harvey Research Institute, Queen Mary University of London, London, UK; 29 Department of Clinical and Movement Neuroscience, Queen Square Institute of Neurology, University College London, Queen Square House, London, WC1N 3BG UK

**Keywords:** Neuronal intranuclear inclusion disease, Hornerin, Repeat expansion disorders

We read with interest the work by Park and colleagues, which attempted to elucidate the composition of neuronal intranuclear inclusions (NIIs), central to the pathology of neuronal intranuclear inclusion disease (NIID) [1]. NIID is a clinically heterogeneous neurodegenerative disorder characterised by these intranuclear eosinophilic ubiquitinated inclusions in both neuronal and non-neuronal cells [2]. Using different proteomic approaches to study compositionally biased regions, which have traditionally been elusive to analysis due to their inherent insolubility, the authors identified hornerin, a serine-rich protein, to be a major component of the inclusions [1].

The molecular aetiology of NIID had remained unresolved for decades since its first pathological characterisation until recently, when a GGC repeat expansion in the 5’UTR of the human-specific *NOTCH2NLC* gene mainly associated with disease in the East Asian population was discovered [3, 4]. This abnormal expansion of GGC repeats has since heralded a new disease entity of polyglycine disorders [5], with evidence for canonical translation of the repeat into a pathogenic polyglycine-containing protein that co-localises with p62-positive NIIs in NIID [6]. However, NIID is genetically heterogeneous, with the GGC repeat expansion in *NOTCH2NLC* being rare in Europeans [7].

Thus, Park and colleagues rightfully assessed NII composition in the post-mortem brain of an individual of European (Finnish) ancestry with juvenile-onset NIID, not associated with the *NOTCH2NLC* repeat expansion [7, 8], to gain further insight into the currently unknown molecular mechanism of disease within European individuals. While hornerin deposits were detected within the inclusions, a heterozygous missense variant in the hornerin (*HRNR*) gene exon 3: NM_001009931.3: c.3023 G > C, p.(Ser1008Thr) was the only variant found on whole exome sequencing, although in silico analysis and a Finnish allele frequency of 0.001748 (within gnomAD v.3.1.2 [9]) deemed it to be unlikely pathogenic.

In order to investigate the genetic basis, extrapolating from the formation of hornerin within the inclusions of the one European case by Park et al. [1], we screened for *HRNR* variants in a large series of ten additional historical cases of pathologically confirmed NIID in patients of European ancestry (confirmed on genotypying), in whom the causative GGC repeat expansion in *NOTCH2NLC* was not found (Table 1) [7]. Furthermore, we also reviewed *HRNR* variants in an European patient with antemortem diagnosis of NIID associated with GGC repeat expansion in *NOTCH2NLC* [7] as well as confirmation in the index case reported by Park and colleagues [7]. We used polymerase chain reaction (PCR) to amplify the 446 base pair region of *HRNR* containing the index variant using conditions by Park et al. [1] followed by Sanger sequencing to review the targeted sequence (Additional file 1: Methods).

**Table 1:** Demographics, clinical presentation and pathological findings of cases of neuronal intranuclear inclusion disease (NIID) examined, with resulting analysis for *HRNR* variants

ID	Age of onset	Age at death	Sex	Family history	Country of origin	Clinical Diagnosis/Presentation pre-biopsy	Main pathological findings and site of pathology	*HRNR* variant	Estimated number of GGC repeats in *NOTCH2NLC*	
									Allele 1	Allele 2
1	17	24	M	Yes	UK	Young-onset parkinsonism and dysautonomia	Widespread neuronal hyaline intranuclear inclusions immunoreactive for ubiquitin and p62 (brain)	No variants detected	21	-
2	33	46	M	Yes	Australia	Slowly progressive motor and sensory neuronopathy with ataxia	Eosinophilic neuronal intranuclear inclusions (brain)	No variants detected	22	28
3	60 s	67	F	No	Australia	Unknown presentation	Eosinophilic intranuclear inclusions in pyramidal cells (brain)	c.3236 G > A, p.(Glu1054Lys), synonymous c.3346 C > T	15	20
4	52	72	F	No	Australia	Slowly progressive primary lateral sclerosis	Cortical neuronal and astrocytic intranuclear inclusions (brain)	No variants detected	15	23
5	11	21	F	Yes (monozygotic twin)	Finland	Ataxia, seizures, and extrapyramidal symptoms	Inclusion bodies in most nerve cell types of central and peripheral nervous systems, as well as in occasional astrocytes	c.3023 G > C, p.(Ser1008Thr) – confirmation of variant found in the same individual by Park et al.[1]	19	22
6	49	62	F	Yes	Spain	Ataxia	Intranuclear hyaline inclusions in neurons and glia in widespread areas of the brain immunoreactive for ubiquitin (brain)	No variants detected	15	25
7	82	84	F	Yes	Spain	Dementia	Intranuclear hyaline inclusions in neurons and glia in widespread areas of the brain immunoreactive for ubiquitin (brain)	No variants detected	16	23
8	26	-	F	No	USA	Unknown presentation	Pathological changes in keeping with NIID (brain)	No variants detected	17	23
9	84	-	M	No	USA	Alzheimer’s disease, ataxia	Intranuclear hyaline inclusions in neurons and glia in widespread areas of the brain (brain)	c.3236 G > A, p.(Glu1054Lys), synonymous c.3346 C > T	15	19
10	69	-	M	No	USA	Diagnosed clinically with NIID	Neuronal intranuclear inclusions (brain)	No variants detected	14	27
11	80	-	M	No	USA	Unknown presentation	Neuronal intranuclear inclusions (brain)	c.3236 G > A, p.(Glu1054Lys), synonymous c.3346 C > T	19	-
12	51	N/A	F	No	Ukraine	Recurrent encephalopathy and migraines	*NOTCH2NLC* repeat expansion positive NIID: Antemortem biopsy contains p62 positive intranuclear inclusions (skin)	No variants detected	19	92–106

All cases were previously investigated for the *NOTCH2NLC* GGC repeat expansion with sizing of the repeat sequence through repeat-primed PCR [7]. NIID cases 1 to 11 were not associated with repeat expansion in *NOTCH2NLC*. Case 12 was the only case found to have a GGC repeat expansion in *NOTCH2NLC* to be associated with NIID, with repeat sizing from Oxford Nanopore Technologies long-read sequencing. Case 5 is the case investigated by Park and colleagues [1, 8]

The previously reported p.(Ser1008Thr) variant in *HRNR* was verified in DNA extracted from heart tissue of the index case using this approach. However, none of the other ten pathologically confirmed NIID cases harboured the same reported *HRNR* variant (Table 1) despite sharing the common characteristic of an absent pathogenic *NOTCH2NLC* repeat expansion and pathological presence of NIIs. Out of these cases, three further European NIID cases diagnosed pathologically through post-mortem brain examination (Cases 3, 9 and 11 in Table 1) were found to have two variants in *HRNR*: a missense variant (c.3236 G > A, p.(Glu1054Lys)) and a synonymous variant (c.3346 C > T) (Fig. 1). However, in silico analysis and prevalent European population frequencies [9] (0.1336 and 0.1362 for the missense and synonymous variants respectively) suggest that these are unlikely to be pathogenic candidates (Fig. 1). As expected, for the patient in which *NOTCH2NLC* repeat expansion was found to be associated with NIID (Case 12), no *HRNR* variants were detected on Sanger sequencing. Moreover, the expression of *HRNR* is not enriched within the central nervous system with low human brain region-specific expression, as exemplified in the Genotype-Tissue Expression (GTEx) project [10].

**Fig. 1:**
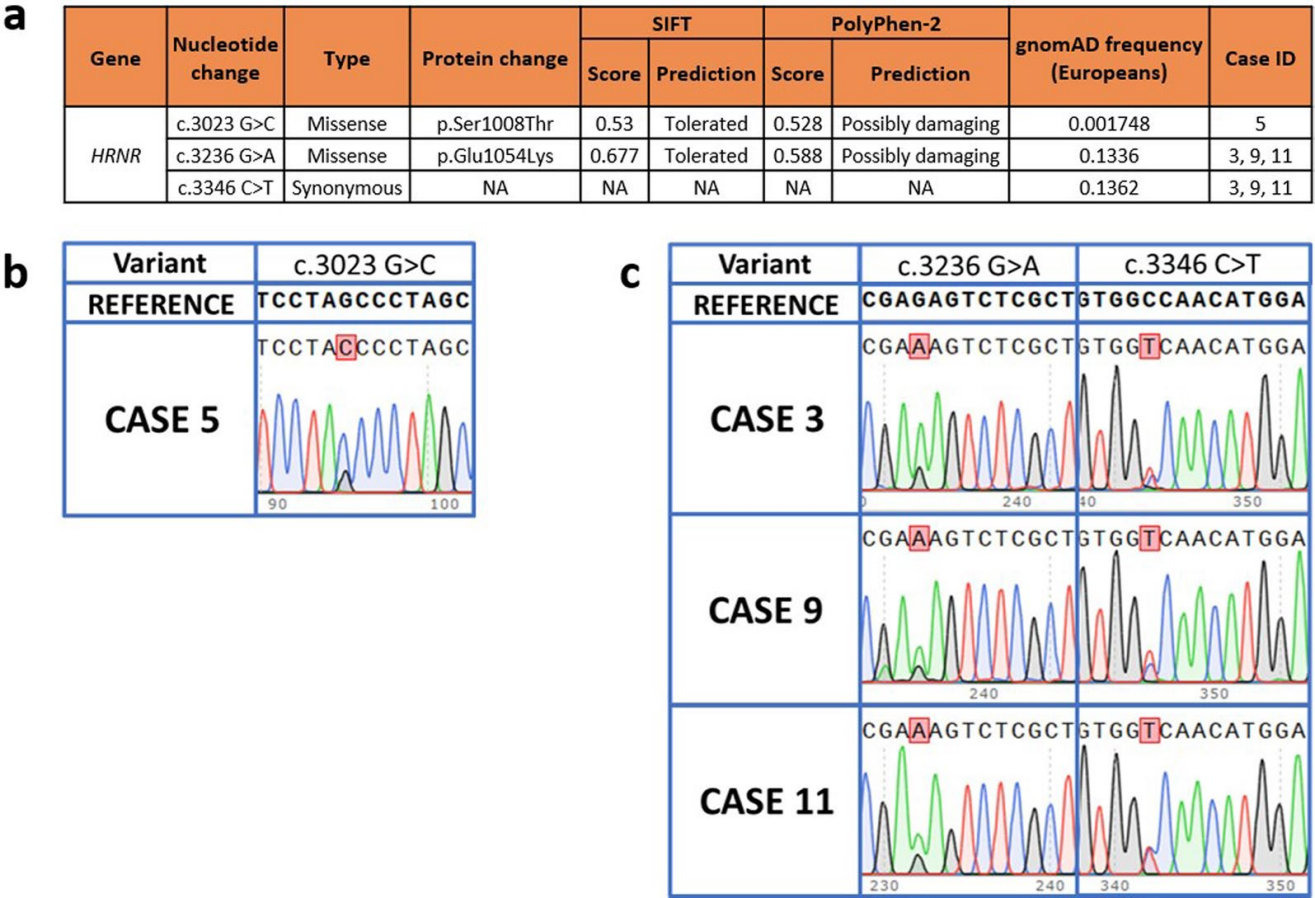
Characteristics of variants detected in *HRNR* in neuronal intranuclear inclusion disease (NIID). **a** Table showing in silico predictions of all variants detected across 12 NIID samples. Sorting Intolerant from Tolerant (SIFT) (https://sift.bii.a-star.edu.sg/) predicts if a substitution at the amino acid level affects protein function with scores ranging from 0 to 1. A variant is predicted damaging to protein function if the score is ≤ 0.05 and tolerated if the score is > 0.05. Polymorphism Phenotyping version 2 (PolyPhen-2) (http://genetics.bwh.harvard.edu/pph2/) is a tool that predicts the possible effect of an amino acid substitution on protein function, with scores ranging from 0 (most probably benign) to 0.999 (most probably damaging). **b** The c.3023 G > C variant detected in Case 5, but not in any other cases, verifies the findings from Park and colleagues[1]. This variant of interest is highlighted in the chromatogram. **c** Missense variant c.3236 G > A and synonymous variant c.3346 c > T found in cases 3, 9 and 11. These variants of interest are highlighted in the chromatogram

Taken together, these findings support those of Park and colleagues, albeit in a larger cohort of *NOTCH2NLC-*negative NIID in patients of European ancestry. The molecular basis of disease in these cases, which are genetically distinct from East Asian NIID cases, is unlikely to be secondary to single nucleotide variation within *HRNR.* It should be noted that while the identification of hornerin as a major component of NIIs in this Finnish case [8] is of interest in providing further molecular insight into the pathogenesis of *NOTCH2NLC* repeat-negative NIID, further direct identification of NII composition in other such molecularly undetermined cases [7] is essential in moving towards establishing the underlying aetiology. The identification of a common genetic explanation for European NIID has thus far remained elusive due to the lack of large pedigrees, a likely complex variant that has eluded conventional sequencing techniques, paucity of antemortem diagnostic clues (as seen in East Asian NIID) and the clinical and genetic heterogeneity of disease. As such, the overarching clue to driving a molecular diagnosis may lie in the accurate pathological characterisation of such disorders, as attempted by Park and colleagues [1], in order to decipher convergent mechanisms for pathogenesis.

### Supplementary Information

**Additional file 1.** Supplementary Methods.

## Data Availability

The datasets used and/or analysed during the current study available from the corresponding author on reasonable request.

## Abbreviations

NIL: Neuronal intranuclear inclusions
NIID: Neuronal intranuclear inclusion disease
PCR: Polymerase chain reaction
