# Supplementary material for: Letter to the editor on: Hornerin deposits in neuronal intranuclear inclusion disease: direct identification of proteins with compositionally biased regions in inclusions by Park et al. (2022)
Source: Acta Neuropathol Commun. 2024 Jan 2;12:2. doi: 10.1186/s40478-023-01706-7 (PMC10759526; doi:10.1186/s40478-023-01706-7)
Supplement: Supplementary file 1 — Additional file 1. Supplementary Methods. [file 40478_2023_1706_MOESM1_ESM.docx]

**Supplementary Methods**

The study was approved by UCL Queen Square Institute of Neurology Institutional Review Board. Tissue and DNA samples from other institutions met approval from local ethics boards. NIID cases were identified from: UK (Queen Square Brain Bank); Spain (FRCB-IDIBAPS Brain Bank Hospital Clinic, Barcelona); Finland; Australia (South Australian Brain Bank and Macquarie University) and USA (Mayo Clinic). DNA extraction from QSBB, Spain, and USA samples of fresh frozen cerebellar tissue was carried out as per Qiagen Gentra Puregene Tissue Kit protocol. For the index Finnish case, DNA was extracted from heart tissue as per Monarch High Molecular Weight DNA Extraction Kit for Tissue protocol.

Polymerase chain reaction (PCR) was designed to amplify the 446 bp region of *HRNR* containing the variant site. PCR mastermix was prepared using FastStart PCR Master (Roche), PCR grade water (VWR) and 5% dimethyl sulfoxide (DMSO, Sigma-Aldrich). Primers used were 5’-CATCTAGGAGCGAACAACATGG-3’ and 5’-ATAGCCAGAAGACTGACTTGAGC-3’. PCR conditions used were provided by Park *et al*. through personal correspondence: initial denaturation at 94 °C for 1 minute, followed by 40 cycles of 94 °C for 30 seconds, 55 °C for 30 seconds and 72 °C for 30 seconds, 72 °C for 7 minutes and 12 °C for 10 minutes. PCR products were run on 2% agarose gel electrophoresis at 100 V for 30 minutes and were visualised under fluorescence light to ensure amplification of the desired region. The PCR products were cleaned up using ExoSAP-IT (Thermo Fisher) and were sent to a commercial company (Source BioScience) for Sanger sequencing. Results were analysed on Snapgene (GSL Biotech LLC) in order to review the nucleotide sequence.
